# Identification of transcriptome signatures and biomarkers specific for potential developmental toxicants inhibiting human neural crest cell migration

**DOI:** 10.1007/s00204-015-1658-7

**Published:** 2015-12-26

**Authors:** Giorgia Pallocca, Marianna Grinberg, Margit Henry, Tancred Frickey, Jan G. Hengstler, Tanja Waldmann, Agapios Sachinidis, Jörg Rahnenführer, Marcel Leist

**Affiliations:** Department of In Vitro Toxicology and Biomedicine, University of Konstanz, Box 657, 78457 Constance, Germany; Department of Statistics, TU Dortmund University, 44139 Dortmund, Germany; Center of Physiology and Pathophysiology, Institute of Neurophysiology, University of Cologne, 50931 Cologne, Germany; Department of Bioinformatics, University of Konstanz, 78457 Constance, Germany; Leibniz Research Centre for Working Environment and Human Factors (IfADo), Technical University of Dortmund, 44139 Dortmund, Germany

**Keywords:** Developmental toxicity, Alternative testing, Transcriptome profiling, Neural crest cells

## Abstract

The in vitro test battery of the European research consortium ESNATS (‘novel stem cell-based test systems’) has been used to screen for potential human developmental toxicants. As part of this effort, the migration of neural crest (MINC) assay has been used to evaluate chemical effects on neural crest function. It identified some drug-like compounds in addition to known environmental toxicants. The hits included the HSP90 inhibitor geldanamycin, the chemotherapeutic arsenic trioxide, the flame-retardant PBDE-99, the pesticide triadimefon and the histone deacetylase inhibitors valproic acid and trichostatin A. Transcriptome changes triggered by these substances in human neural crest cells were recorded and analysed here to answer three questions: (1) can toxicants be individually identified based on their transcript profile; (2) how can the toxicity pattern reflected by transcript changes be compacted/dimensionality-reduced for practical regulatory use; (3) how can a reduced set of biomarkers be selected for large-scale follow-up? Transcript profiling allowed clear separation of different toxicants and the identification of toxicant types in a blinded test study. We also developed a diagrammatic system to visualize and compare toxicity patterns of a group of chemicals by giving a quantitative overview of altered superordinate biological processes (e.g. activation of KEGG pathways or overrepresentation of gene ontology terms). The transcript data were mined for potential markers of toxicity, and 39 transcripts were selected to either indicate general developmental toxicity or distinguish compounds with different modes-of-action in read-across. In summary, we found inclusion of transcriptome data to largely increase the information from the MINC phenotypic test.

**Electronic supplementary material:**

The online version of this article (doi:10.1007/s00204-015-1658-7) contains supplementary material, which is available to authorized users.

## Introduction

Stem cell-based in vitro test systems offer new possibilities to explore toxicological hazard directly on relevant and non-transformed human cells. This novel approach particularly benefits some complex toxicological areas, such as developmental toxicity, that have not been accessible in the past to human-relevant testing.

Besides the three germ layers forming the different tissues and organs, a so-called fourth layer plays a role in this complex stage: the neural crest (NC). The NC is a multipotent migratory cell population that emerges from the dorsal aspect of the neuronal tube in the early phases of development and gives rise to a multitude of different cell types, supporting the formation of cartilage, bone, connective tissue of the face, but also neurons, glial cells, melanocytes and cardiomyocytes (Huang and Saint-Jeannet 2004). A large percentage of developmental disorders (e.g. congenital heart defects, orofacial clefts, Hirschsprung’s disease) are caused by NC cells (NCC) deficit, and these often correlate with neural tube defects. This kind of alterations can be induced by genetic factors (Lee et al. 2009) or exposure to pharmaceuticals (e.g. valproic acid, Fuller et al. 2002) and pesticides (e.g. triadimefon, Menegola et al. 2000).

In the field of in vitro DNT testing, a new approach is being explored, based on the use of human cells and on the identification and modelling of distinct key biological processes representing possible targets of a toxicant. The effects of a toxicant may be described as the set of alterations of endpoints in such test systems. Examples for such test systems are the neurite outgrowth assay (Krug et al. 2013a) and the neural crest cell migration (MINC) assay (Zimmer et al. 2012; Dreser et al. 2015). These two exemplary assays are based on the quantification of a functional endpoint (neurite outgrowth and number of migrated cells) in relevant biological systems (LUHMES-derived neurons and human embryonic stem cell (hESC)-derived NCC).

Other test systems explore, e.g., changes of neural differentiation (Balmer et al. 2012), gliogenesis (Fritsche et al. 2005), myelination (Zurich et al. 2000, 2002) or synaptogenesis (Harrill et al. 2011). Usually, the test systems also address defined developmental stages (Stummann et al. 2009; van Dartel et al. 2009; Zimmer et al. 2011).

Therefore, in vitro testing strategies usually require a battery of tests (Leist et al. 2014; Bal-Price et al. 2015; Rovida et al. 2015) in order to cover most of the key biological and molecular events.

Different types of test batteries have been recently presented: they may consist of molecular-based assays, like in the ToxCast program (Sipes et al. 2011; Padilla et al. 2012) or cell-based assays, as, for example, in the ReProTect (Schenk et al. 2010) or ChemScreen (van der Burg et al. 2014, 2015) projects.

A further step into this direction was taken by the ESNATS consortium with the establishment of a stem cell-based test battery (Zimmer et al. 2014). This testing approach was designed in a modular way to allow any interested user to join in and to add their test system as well as the data generated from it. The ESNATS test battery has some features that distinguish it from earlier approaches, the two most important ones referring to the test chemicals selection and to the follow-up procedure of positive screen results (hits).

The usual approach of test chemical selection for new assays (Leist et al. 2010; Crofton et al. 2011; Kadereit et al. 2012) is based on the compilation of chemicals with predefined activity (i.e. known positives and negatives) to be used as gold standard to calibrate the assay. This approach is difficult for in vitro test systems of developmental toxicity for two reasons. First, only few such gold-standard compounds are known from reliable in vivo studies or human epidemiology; second, also for known compounds, it is often not clear how they are expected to behave in an in vitro system. To get out of this dilemma, other approaches of assay validation and selection of initial test compound sets have been suggested (Leist et al. 2012a; Hartung et al. 2013; Smirnova et al. 2014). In essence, such alternative strategies comprise two steps: first, the test system undergoes mechanistic validation on the basis of tool compounds that verify that the expected biochemical features and signalling pathways are represented by the system; second, once trust in the biological relevance of the systems is established, a broad set of interesting compounds can be tested. These chemicals are then classified as potentially hazardous (or not), based on the outcome of the screen (Behl et al. 2015; Pei et al. 2015). Thus, this approach takes the opposite direction from the classical approach. The advantage of this strategy is that a broad range of compounds is characterized for a potential developmental toxicity hazard, ideally across multiple test systems (test battery). When chemicals are identified as developmental toxicity hazard, they can be used as gold standards for further test system establishment, and consequently, the pool of well-characterized compounds required for test system set-up grows. The ESNATS test battery (Zimmer et al. 2014) and a 76-compound library of the national toxicology program of the USA (Pei et al. 2015) were set up with this goal in mind.

The test compound list of the ESNATS battery comprised 28 substances, including biologics, pharmaceuticals and environmental toxicants. The background data of these compounds were extensively documented, for instance concerning general cytotoxic potency, chemical and pharmacokinetic data, as well as further relevant findings retrieved by literature data mining. In 2014, a first screening was performed using one of the assays included in the test battery, i.e., by the migration inhibition of neural crest cells (MINC) assay. This test was included in the ESNATS test battery project (as UKN2 system) to cover the developmental stage of neural crest with a cell function-specific endpoint (migration; Zimmer et al. 2012). The MINC screening led to the identification of 11 hits, comprising all of the environmental chemicals (positive controls) and some little-characterized pharmaceuticals. In contrast to some other screening algorithms, the ESNATS test battery scheme consists of an extensive part of hit follow-up to further characterize the chemicals and their effect in the test system. This is considered an important activity towards the overall objective of identifying new potential gold-standard developmental toxicants on the basis of their in vitro effects. One of the follow-up activities firmly anchored in the test battery scheme is the full characterization of transcriptome changes triggered by the hits. In this context, it is important to note that the hits of the MINC assay are chosen because of their functional effects in the test system, i.e. because they inhibit a cell function considered to be essential for normal human development. This starting point provides thus a phenotypic anchoring of the transcriptome data to be obtained.

In the present study, we selected the six most robust and novel hits for further characterization by transcriptome profiling. They included the chemotherapeutics geldanamycin and arsenic trioxide, the flame-retardant PBDE-99, the pesticide triadimefon and the histone deacetylase inhibitors valproic acid and trichostatin A. We performed a whole-transcriptome analysis to detect changes triggered by these substances in human neural crest cells. Three main questions were asked: (1) can transcriptome profiles of NCC be used to identify DNT compounds; (2) how can transcriptome information be reduced to toxicological profiles; (3) which are the possible approaches to identify candidate biomarkers from transcriptome profiles.

To answer these questions, the present study included a blind study, probing the predictivity of compound-transcriptome pattern matching. Furthermore, we developed new visualization tools to display the toxicity pattern of each substance based on transcriptome data. Finally, we proposed two approaches to prioritize and select candidate biomarkers which led to the identification of 39 transcripts to be further explored as NCC toxicity indicators.

## Materials and methods

### Cell culture and neural crest differentiation

The reporter hES cell line H9-Dll1 (GFP under Dll1 promoter) was provided by Mark Tomishima from the Memorial Sloan Kettering Cancer Centre (MSKCC, NY, USA). Import of the cells and all experiments was carried out according to German legislation under the licence number 1710-79-1-4-27 of the Robert-Koch Institute.

H9-Dll1 cells were maintained on mouse embryonic fibroblasts (MEFs) in DMEM/F12 (Gibco) medium containing 20 % of serum replacement, HEPES (1 M, Gibco), l-gluthamine (Glutamax, Gibco), non-essential amino acids (MEM NEAA, Gibco), beta mercaptoethanol (Gibco) and basic fibroblast growth factor (10 ng/ml, Invitrogen).

Differentiation of hESC into neural crest cells (NCC) was initiated on Mitomycin C-treated murine bone marrow-derived stromal MS5 cell line and continued as described in Zimmer et al. (2012).

### Chemical exposure during NCC migration

hESC-derived NCC were exposed for 48 h to non-cytotoxic concentration of different NCC migration-inhibiting substances in N2 medium containing EGF (20 ng/ml) and FGF2 (20 ng/ml). Six compounds were used: geldanamycin (16 nM, Selleckchem), arsenic trioxide (1 µM, Sigma-Aldrich), thricostatin A (TSA, 10 nM, Sigma-Aldrich), valproic acid sodium salt (VPA, 250 µM, Sigma-Aldrich), triadimefon (100 µM, Bayer Crop Science) and pentabromodiphenyl ether (PBDE-99, 15 µM, Clickchem). Two different solvent control groups were also produced: NCC were exposed to 0.04 % DMSO or simply to N2 medium for 48 h. Finally, a third control group (exposed to N2 medium only) was added specifically as control of arsenic trioxide, since the testing of this substance was performed not in parallel with the other compounds.

### Affymetrix gene chip analysis

Samples of ≥5 × 10^6^ cells were collected using RNA protect reagent from Qiagen. The RNA was quantified using a NanoDrop N-1000 spectrophotometer (NanoDrop, Wilmington, DE, USA), and the integrity of RNA was confirmed with a standard sense automated gel electrophoresis system (Experion, Bio-Rad, Hercules, CA, USA). Analysis was then performed as described earlier (Krug et al. 2013b) using Affymetrix chip-based DNA microarray (Human genome U133 plus 2.0 arrays) with all standard quality control procedures.

### Biostatistics

The microarray data analysis (extrapolation and normalization of the array sets) was performed using the statistical programming language R-version 3.1.1 as described in Waldmann et al. (2014). For the normalization of the entire set of Affymetrix gene expression arrays, the extrapolation strategy (RMA+) algorithm (Harbron et al. 2007) was used that applies background correction, log_2_ transformation, quantile normalization and a linear model fit to the normalized data in order to obtain a value for each probe set (PS) on each array. As reference, the normalization parameters obtained in earlier analyses (Krug et al. 2013b) were used. After normalization, the difference between gene expression and corresponding controls was calculated (paired design). Differential expression was calculated using the R package limma (Smyth et al. 2005). Here, the combined information of the complete set of genes is used by an empirical Bayes adjustment of the variance estimates of single genes. This form of a moderated *t* test is abbreviated here as ‘limma *t* test’. The resulting *p* values were multiplicity-adjusted to control the false discovery rate (FDR) by the Benjamini–Hochberg procedure (Benjamini 1995). As a result, for each compound a gene list was obtained, with corresponding estimates for log fold changes and *p* values of the limma *t* test (unadjusted and FDR-adjusted).

Transcripts with FDR-adjusted *p* values of ≤0.05 and fold change values of ≥1.8 were considered significantly deregulated and defined as differentially expressed genes (DEG).

### Data display: heatmap and principal component analysis

The software R (version 3.1.1) was used for all calculations and display of PCA and heatmaps. Principal component analysis (PCA) plots were used to visualize expression data in two dimensions, representing the first two principal components. The percentages of the variances covered are indicated in the figures. Heatmaps were used to visualize matrices of gene expression values.

The hierarchical clustering analysis was performed as previously described (Krug et al. 2013b). Complete linkage was used as agglomeration rule for the clustering analysis. Distances based on the Euclidean distance measure were used to group together transcripts with similar expression patterns across samples (rows of the heatmap). Then, expression values within each row were normalized as *Z*-factors and colour-coded accordingly. Colour encodes the magnitude of the values as *z* score, ranging from blue (low) to yellow (high).

### Support vector machine-based classification

A support vector machine algorithm with linear kernel was used for the discrimination between two data sets: a training group composed of three biological replicates and a testing group composed of two biological replicates (with compounds blinded to the experimenter) using the same set of compounds. Both groups were normalized to the respective controls; i.e. the difference between gene expression and corresponding controls was calculated (paired design). Geldanamycin, PBDE-99 and triadimefon had common controls, valproic acid (VPA) and trichostatin A (TSA) were assigned to the same set of controls, and arsenic trioxide had its own set of controls. After subtracting controls, the number of variables was reduced to the 100 probe sets with highest variance within the training set. Then, in a second step, the hyperparameters for optimizing the decision boundary between the known training compounds were determined (using a grid search over supplied parameter ranges). These parameters were then used to generate the classification model to predict for the blinded testing sample the probabilities to belong to the known training compounds. For multiclass classification with more than two classes, first in a ‘one-against-one’ approach, all possible binary classifiers were trained and corresponding probabilities were calculated from a logistic regression as described in Rempel et al. (2015). Then, a posteriori class probabilities for the multiclass problem were obtained using quadratic optimization.

### Gene ontology (GO) and KEGG pathway enrichment analysis

The gene ontology group enrichment was performed using R-version 3.1.1 with the topGO package (Alexa et al. 2006) using Fisher’s exact test, and only results from the biological process ontology were kept. Here, again the resulting *p* values were corrected for multiple testing by the method of Benjamini–Hochberg (Benjamini 1995).

The KEGG pathway analysis was performed using the R package ‘hgu133plus2.db’ (Carlson 2015). Probesets are mapped to the identifiers used by KEGG for pathways in which the genes represented by the probe sets are involved. The enrichment was then performed analogous to the gene ontology group enrichment using Fisher’s exact test.

Up- and downregulated differentially expressed genes were analysed separately for each treatment. Only GO classes and KEGG pathways with a BH-adj. *p* value ≤0.05 were considered significant.

### Toxicity pattern visualisation

ToxPi diagrams as developed in the ToxCast project (Kleinstreuer et al. 2011; Filer et al. 2014) were constructed using a Web-based user interface (Reif et al. 2013). For this purpose, the numbers of DEG of overrepresented GO groups and KEGG pathways were normalized to the highest respective values for each category across all compounds (359 for DEG, 373 for GO classes and 17 for KEGG pathways). The ToxPi score was calculated over the six parameters used for ToxPi construction, with double weight given to KEGG and GO versus DEG.

GO classes and KEGG pathway terms were included only when ≥3 differentially expressed genes were found in the enriched term or (for small groups) when ≥50 % of the genes belonging to the GO/KEGG group were found to be significantly altered in our study.

### GO superordinate classes distribution

The gene ontology group enrichment was performed as described above. Up- and downregulated differentially expressed genes were analysed together for each treatment. Only GO classes with a BH-adj. *p* value ≤0.05 were considered significantly enriched. Classic and elim methods (described in Alexa et al. 2006) were both used: classic method was chosen for geldanamycin treatment, while elim method was used for the analysis of the other compounds. The elim algorithm iteratively removes the genes mapped to significant GO terms from more general (higher level) GO terms, whereas the classic algorithm neglects the local dependencies between GO terms in its calculations.

Enriched GOs were then assigned to superordinate cell biological processes as already described in Waldmann et al. (2014) and distributed in six classes: migration/adhesion, metabolism, differentiation, signalling, stress response and others. The migration class includes migration- and adhesion-related GO classes; stress response class includes cell death-, extracellular stressor-, inflammation/immunity-related GO classes; signalling class consists of cell receptor activity-, second messenger (cAMP, cGMP, Ca2+) metabolism-, kinase modification-related GO classes. Metabolism class comprise all GO classes covering metabolism activity; differentiation class includes cell differentiation-related GO classes; ‘other’ class covers all the others: not otherwise classified GO classes.

### Biomarker quality control

To evaluate how the here-chosen set of 39 biomarkers (set_*A*) compared to random combinations of 39 genes (set_*X*_*i*_; *i* = 1–1,000,000), a simple metric for the separation power (controls vs six test compounds) of biomarker sets was developed and expressed as separation units (SU). The distribution of these SU was then determined by bootstrapping (one million samples), and the relative position of set_*A* in this distribution was determined. The metric is based on the following procedural steps: (1) for each of the 54,675 probe sets (PS), a *T* score was calculated for comparison of one of the six test compounds with the control (with *n* = 5 for controls and test compound samples); i.e. six *T* values were obtained for each PS [one for each of the compounds geldanamycin (GA), triadimefon (TDF), VPA, TSA, arsenic trioxide (As_2_O_3_) and PBDE]; (2) only the PS with at least one compound showing a difference from control with *p* < 0.05 were considered in further steps; (3) the *T* scores of the group of PS assigned to the same gene were averaged; (4) the genes were ranked according to their *T* scores. This was done individually for each test compound; then the ranks were normalized to a ranking value (‘*r’*; values from 0 to 1, where *r* = 0 corresponds to low *T* score [low grade of separation between control and exposure) and *r* = 1 to high *T* score (high grade of separation)]; (5) the rank values of 39 biomarker genes (of set_*X*_*i*_ or set_*A*) were averaged to give a ‘biomarker separation rank’ for one given compound; (6) the six biomarker separation ranks for the toxicants were assumed to define a vector in six-dimensional space, indicating the distance of toxicants from the control. The length of this vector was used as metric for the SU:

$$ {\text{SU}} = \sqrt {\left( {\bar{r}_{\text{GA}}^{2} + \bar{r}_{\text{TDF}}^{2} + \bar{r}_{\text{PBDE}}^{2} + \bar{r}_{\text{TSA}}^{2} + \bar{r}_{\text{VPA}}^{2} + \bar{r}_{{{\text{As}}_{2} {\text{O}}_{3} }}^{2} } \right)} . $$

The length of this vector was in the range 0–2.45. The randomly chosen set_*X*_*i*_ had an average SU of 1.256 ± 0.081, and the set_*A* was at 1.75 (i.e. >6 SD larger than the average).

## Results

### Data structure of transcriptional changes induced in neural crest cells exposed to migration-inhibiting concentrations of test battery hits

**Fig. 1:**
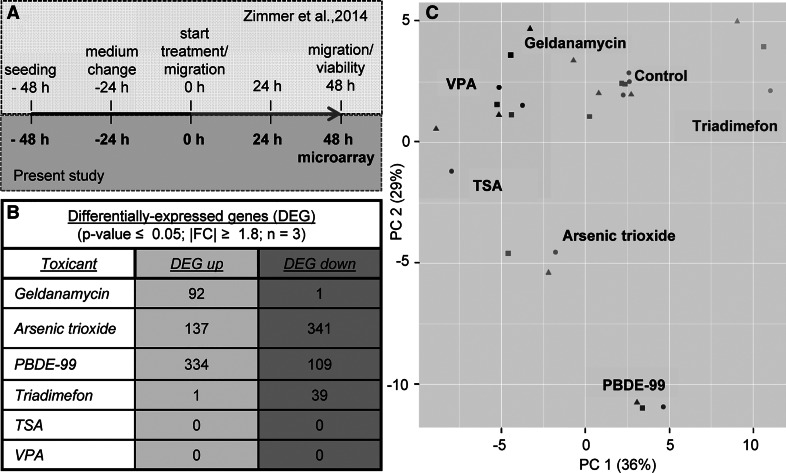
Experimental design and transcriptome data structure of test battery hits. **a** Sampling for microarray analysis was performed in neural crest cells after 48-h exposure (*red arrow*) to non-cytotoxic and migration-inhibiting concentrations of six test battery hits (geldanamycin, arsenic trioxide, PBDE-99, triadimefon, TSA and VPA), as identified by the method and the data published in Zimmer et al. (2014). **b** The differentially expressed genes (DEG) were identified for each condition: the number of up- (DEG up) and downregulated genes (DEG down) are shown in the table (details can be found in the supplementary material; *p* values were FDR-corrected). **c** Principal component analysis (PCA) was performed, based on the 100 transcripts with highest variance, and a 2D plot was generated to display the transcriptome data structure across compounds and experimental replicates. *Each point* represents one experiment (= data from one microarray), where the chemical label supported by the *colour coding* indicates the compound and the form of the *data points* indicates the replicate. The percentages of the variances covered are indicated on the *axes* (colour figure online)

To explore the effects of neural crest toxicants on the transcriptome, six compounds were selected that had been shown earlier to inhibit migration of human neural crest cells (NCC) in the MINC assay (Zimmer et al. 2014; Dreser et al. 2015). They comprised the heat-shock protein modifier and new chemotherapeutic lead compound geldanamycin (GA), the chemotherapeutic agent and environmental toxicant arsenic trioxide (As_2_O_3_), the brominated flame-retardant PBDE-99, the triazole pesticide triadimefon (TDF) and the histone deacetylase inhibitor (HDACi) trichostatin A (TSA) and valproic acid (VPA). To allow comparisons amongst the compounds, NCC were exposed for 48 h to each of the toxicants at their respective highest non-cytotoxic concentration and to solvent controls. Then, mRNA was prepared from three different cell lots and used for gene expression analysis by Affymetrix microarray technology (Fig. 1a).

After initial data processing and quality controls, the gene expression data were used to determine the differentially expressed genes (DEG). The DEG for each toxicant were defined here as the group of microarray probe sets (PS), which differed significantly from negative controls (FDR-adjusted *p* value ≤0.05) and showed expression level changes (fold change (FC)) of ≥1.8 or ≤0.55. On this basis, the toxicant effects could be roughly categorized: strong effects on the transcriptome were observed for As_2_O_3_ (478 DEG) and PBDE-99 (443 DEG), a medium effect was detected for GA (93 DEG) and triadimefon (40 DEG), while no effect was observed for TSA and VPA (0 DEG). Moreover, we observed three different types of response, concerning the direction of gene regulations: a predominant upregulation (ratio between upregulated DEG (DEG up) and downregulated DEG (DEG dw) ≥10 (e.g. GA); a predominant downregulation, with a ratio between DEG up and DEG dw of ≤0.1 (e.g. triadimefon); and mixed, bidirectional regulations (e.g. As_2_O_3_ and PBDE-99) (Fig. 1b).

To visualize the different gene expression profiles across all compounds and replicates, a principal component analysis (PCA) was performed, based on the 100 PS with highest variance amongst the samples. Plotting of the first two principal components showed that the replicates of each compound clustered closely together, while the compounds clearly separated from the nine controls and from one another. As expected, the two ‘strong-effect’ compounds, As_2_O_3_ and PBDE-99, showed the most distinct separation from controls and from one another. Notably, also HDACi (TSA and VPA) separated clearly from controls, but they could not be separated from each other (Fig. 1c). This separation effect in the PCA (despite the absence of DEG for HDACi) was due to the combined use of an ensemble of 100 PS selected by highest variance amongst samples, instead of a gene-by-gene comparison.

In conclusion, the initial analysis of the microarray data showed that the quality of the gene expression data sets was high enough for further exploration and that different migration inhibitors appeared to trigger distinct signatures of gene expression changes in NCC.

### Computational toxicant identification based on correlation between transcriptome data sets

**Fig. 2:**
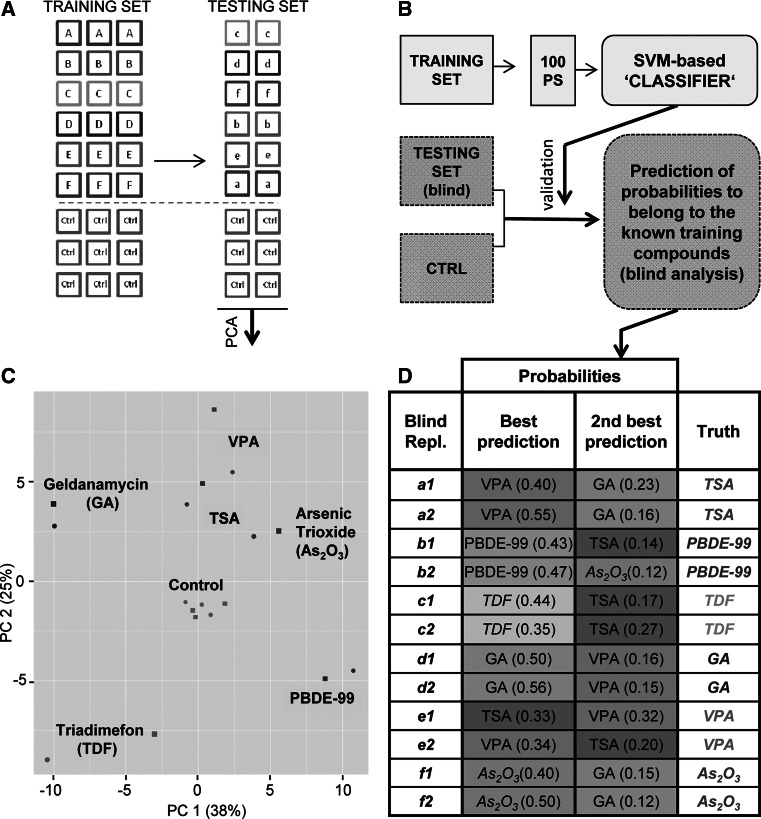
Correlation analysis between ‘training set’ and blind ‘testing set’ data. **a** Two data sets were generated: a training set, based on the data presented in Fig. 1 (*boxes with capital letters*, *n* = 3), and a testing set, based on data obtained by additional two replicates (with compounds blinded to the experimenter), using the same set of compounds (*boxes with small letters*, *n* = 2). Both groups were normalized to the respective controls (Ctrl, *orange boxes*). **b** A PCA plot based on the 100 transcripts with highest variance was generated to display the structure of the transcriptome data of the testing set along the first two principal components. **c** The 100 probe sets with highest variance (‘100 PS’) within the training set were identified. Then, a classifier was built using the support vector machine (SVM) approach (see methods). Finally, the probabilities of the blinded testing samples to belong to the known training compounds were predicted. **d** The best and second best predictions, based on a support vector machine approach (indicated as relative probability in the *brackets*), are listed for each blind replicate (*first column*). The real identity of the samples (truth) is indicated in the last column. For instance, the highest probability (50 % likelihood) for blind sample d1 was obtained for geldanamycin (GA), and the second highest (16 % likelihood) was for VPA. The unblinding of the sample revealed it to be GA (colour figure online)

While the initial analysis suggested that a post hoc separation of toxicants was possible to some extent, we were interested in the predictive power of the transcriptome analysis. For this purpose, the data sets obtained initially were used as training set of a classification rule, while new data for each compound (*n* = 2 additional experiments) were obtained as testing set (Fig. 2a). The ‘testing set’ experiments were performed in a way that it was known to the experimenter, of which samples were negative controls, while the identity of the toxicants to be tested was blinded.

To obtain an overview over the new data, a PCA of the testing set microarrays was performed after ‘unblinding’ of the data to the person doing the analysis. The results showed that the structure of the collected transcriptome data resembled the one of the training set; i.e. all toxicant samples separated ‘visually’ from the six controls, and the couples of toxicant replicates were closer to one another than to other data points (Fig. 2b).

In the next step, the 100 probe sets with highest variance within the training set were used for the classification analysis, in an attempt to identify the treatment of each of the blinded samples of the testing set. For this purpose, a support vector machine approach was used to discriminate between the six different compounds. The training set composed of three biological replicates was used to generate the classifier which includes (1) the selected probe sets, (2) the optimized hyperparameters of the model and (3) the classification rule for belonging to a particular compound. The classifier was then applied to obtain for each blinded testing sample the probabilities to belong to the known training compounds. The probabilities were then sorted in descending order, and the training set condition yielding the highest probability of affiliation was taken as ‘best prediction’ for the corresponding testing set data (Fig. 2c).

Nine out of twelve blind samples were correctly predicted (75 % predictivity); i.e. the correct compound was assigned to the respective microarray. All the ‘wrongly’ predicted samples belonged to the HDACi group, and predictions were correct within the group. If prediction of an HDACi was accepted as correct prediction for VPA or TSA, the overall predictivity of the test was 100 % (Fig. 2d).

The predictivity of the classifier was not affected by the separation of the five microarrays per compound into training and testing set: a simulation study was performed by random selection of the replicates belonging to the training or testing data sets: 1000 different combinations were analysed, and for all compounds, with exception of the HDACi group, ~100 % correct predictions were observed (Fig. S1A).

The prediction may suffer from a skewing of our compound collection, as the two HDAC inhibitors formed a group amongst themselves and thus led to an overrepresentation of a defined toxicological mechanism within the group. For this reason, we performed a second set of analyses after exclusion of TSA from the compound set. In this new scenario, we reached 100 % predictivity, with 10 of 10 correct predictions (Fig. S1B).

The outcome of this small blind-testing study suggests that microarray data may be useful to assign unknown compounds to predefined groups of compounds, for instance, to obtain some initial toxicological information or for biology-supported read-across.

### Characterization of toxicant-induced transcriptome profiles

**Fig. 3:**
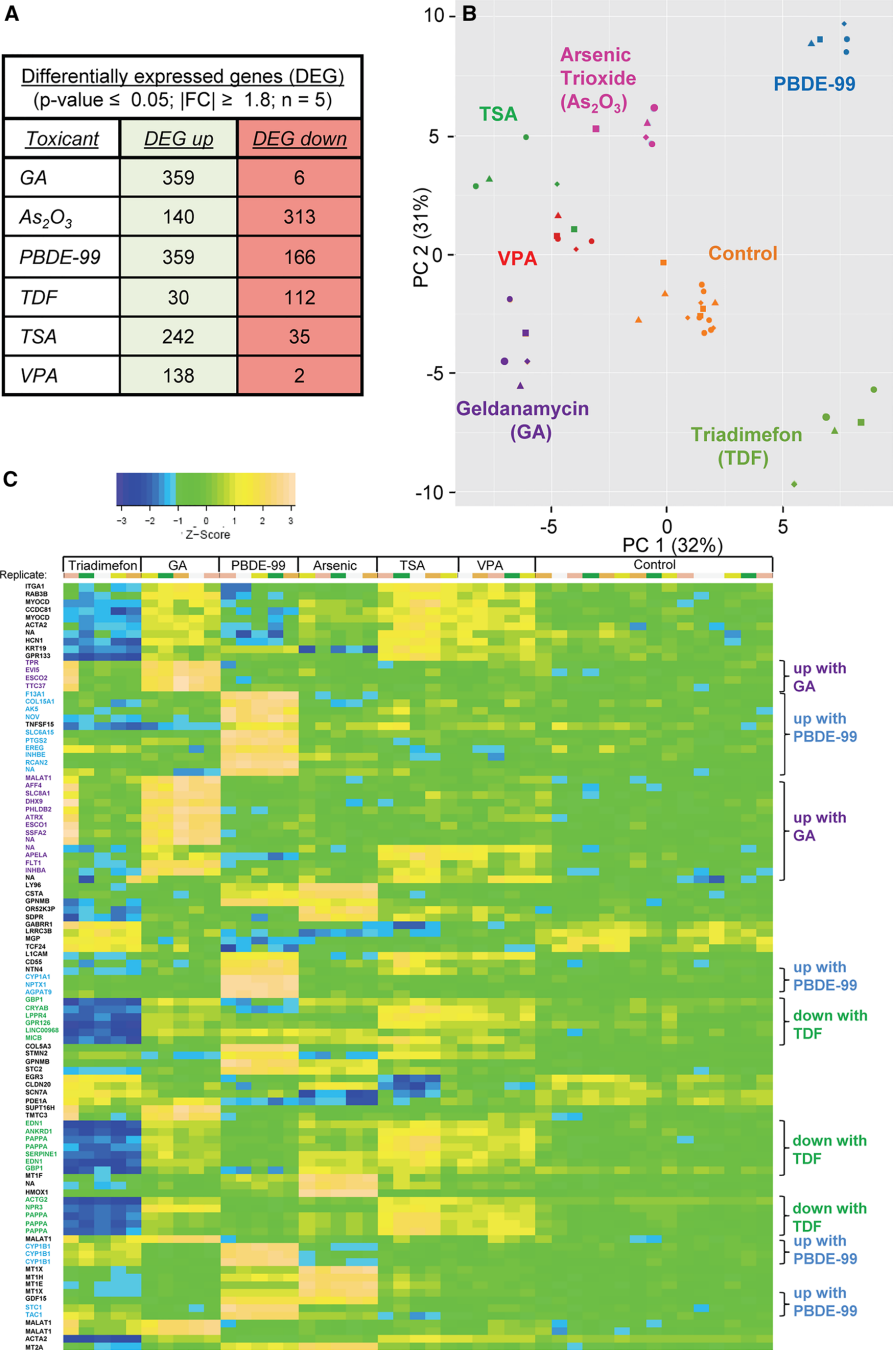
Overall transcriptome changes based on pooled data sets. **a** Training and testing expression data sets were analysed together to identify overall transcriptome changes. The number of differentially expressed genes significantly up- or downregulated is listed in the table for each condition (detailed data are shown in supplemental material). **b** The transcriptome data structure was displayed as principal component analysis (PCA) 2D plot showing the first two principal components. Percentages of covered variances are indicated on the axes. **c** The transcriptome data were represented as heatmap indicating the gene expression values of the top 100 probe sets with the highest variability amongst the compounds. Expression values of the individual genes were transformed to *z* scores (*along rows*). *On the right* some exemplary gene groups are indicated for guidance (e.g. genes upregulated specifically by geldanamycin)

To obtain more in-depth insight into the transcriptome changes induced by each compound, the training and testing set data were combined and analysed together for DEG. The increase in the statistical power (due to the increase in replicate numbers; *n* = 5) allowed the detection of a higher number of DEG. For As_2_O_3_ (453 DEG) and PBDE-99 (525 DEG) treatments, the increase was rather moderate, and for GA (365 DEG; nearly all upregulations) and triadimefon (142 DEG; mixed regulation pattern), the increase was substantial. The increased sensitivity of the analysis was particularly evident for TSA (277 DEG) and VPA (140 DEG), which mainly led to gene upregulation (Fig. 3a). The new PCA plot over all conditions (15 controls *plus* 6 × 5 toxicant samples) showed that all compounds separated from controls and from one another, if the HDACi TSA and VPA were considered as one group (Fig. 3b).

The differential effects of the toxicants on gene expression were also evident from a heatmap display that shows the relative expressions of the 100 PS with largest variability. This allowed some first insight on the level of individual genes. For example, a group of PS (*n* = 13) upregulated by geldanamycin only comprised the three cell cycle controllers such as ESCO1, ESCO2 (*N*-acetyltransferases involved in establishment of sister chromatid cohesion), MALAT1 (Tripathi et al. 2013; Yang et al. 2013) and ATRX (Berube et al. 2000). Other examples are the group of PS who were specifically upregulated upon PBDE-99 treatment, such as the cytochromes CYP1A1 and CYP1B1 or tachykinin, or the 17 genes downregulated only by triadimefon (e.g. the inflammation-related factors MICB, endothelin and GBP1; Fig. 3c).

**Fig. 4:**
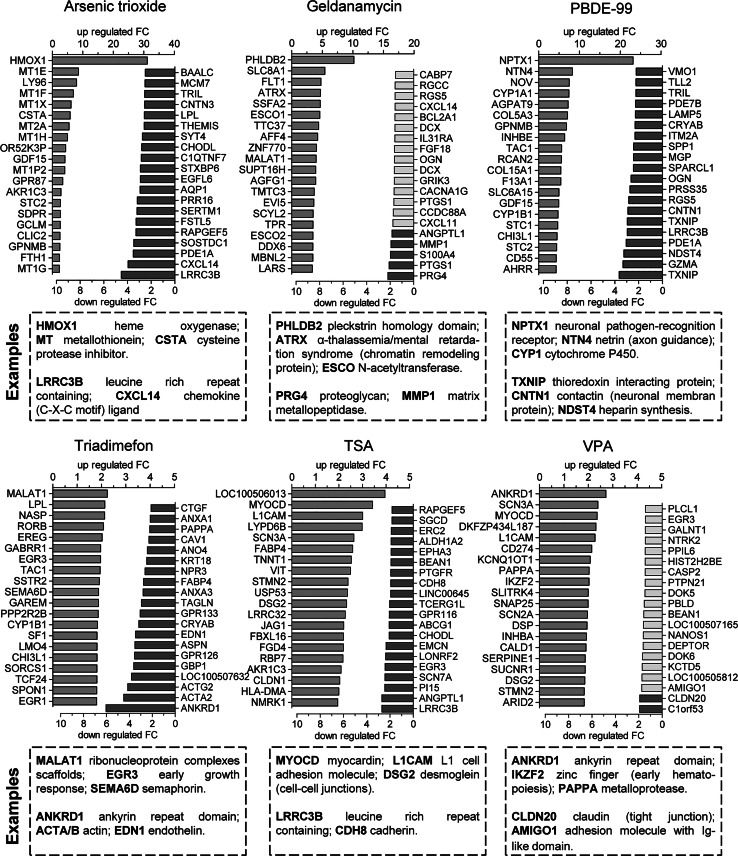
Selection of the 20 most significant up- and downregulated genes for hit compounds. The differentially regulated genes were identified for six hit compounds and sorted according to their *p* value. The top 20 upregulated (*green*) and downregulated (*red*) genes for each condition are shown as *bar graphs* indicating the fold change (FC). Genes regulated with a FC below the threshold of 1.8 are indicated in *light red*. Few example genes were chosen according to their toxicological/pathophysiological interest level according to the literature. This biased selection is displayed only as initial rough overview and food-for-thought for later marker selection (colour figure online)

Encouraged by this visual exploration, the gene expression changes were explored quantitatively on the PS level. The DEG for each toxicant were sorted according to their *p* values and the top 20 up- and downregulated genes were selected. This approach focussed on the statistically most important regulations by each toxicant versus control conditions, without taking into account whether other toxicants affected the same gene (Fig. 4).

Arsenic trioxide affected many heavy metal-induced genes (upregulation of different metallothioneins (MT) isotypes; 30-fold upregulation of heme oxigenase-1), and it upregulated the protease inhibitor cystatin-A, similar to the situation observed in human T cells after treatment with arsenic (Shao et al. 2014). PS strongly downregulated by arsenite comprised CXCL14, a chemokine which is known to play a role in cell migration, and LRRC3B, a gene whose cord blood leucocyte DNA methylation pattern was found altered in a cohort of newborns prenatally exposed to arsenic in water (Rojas et al. 2015).

The exposure to geldanamycin led to a 20-fold upregulation of PHLDB2. The product of this pleckstrin homology domain gene, also known as LL5β, is a protein implicated in migration and tumour cell invasion, by stabilizing of the protrusive activity at the cell front (Astro et al. 2014). Downregulations by geldanamycin were only moderate, and they comprised, e.g., the metalloproteinase MMP1 and the cell matrix proteoglycan PRG4.

Amongst the genes upregulated by PBDE-99, we identified neural pentraxin (NPTX1; ~20-fold upregulated), whose cognate protein exclusively localizes to the nervous system. Moreover, extracellular matrix factors involved in axon guidance were affected, like netrin 4 (NTN4) or the collagens, COL5A3 and COL15A1. The downregulated genes comprised the thioredoxin-interactin protein (TXNIP), whose downregulation was also observed in HUVEC (Kawashiro et al. 2009) and H295R adrenocortical carcinoma cells (Song et al. 2009) after exposure to polybrominated diphenyl ethers. Other examples of downregulations are contactin 1 (a neuronal membrane protein with functions in cell adhesion and the formation of axon connections in the developing nervous system) or NDST4, an enzyme involved in extracellular matrix (heparin) synthesis.

In triadimefon-treated cells, upregulations were very moderate. Amongst the most downregulated genes, we identified the ankyrin repeat domain (ANKRD1) as well as *α* and *β* actin. Moreover, the downregulated DEG comprised endothelin 1, which is involved in neural crest patterning (Pla and Larue 2003). The attenuated activity of this gene may be related to the neural crest toxicity of the pesticide (Di Renzo et al. 2011; Menegola et al. 2005).

HDACi upregulated, e.g., myocardin, similarly as observed in forebrain precursor cells (Balmer et al. 2014), the cell adhesion molecule (L1CAM) and desmoglein, a cell–cell junction glycoprotein. Some of the top downregulated genes (LRRC3B and cadherin-8) were similar to those downregulated by HDACi in central neural precursors (Balmer et al. 2014).

We also observed deregulation of a particular group of genes linked to neurocristopathies, a specific class of pathologies deriving from NCC deficits. Amongst the genes known to be linked to neurocristopathies, three groups were also affected by toxic chemicals: first, endothelin and its receptors (EDN1 and EDNRB; Kurihara et al. 1994), the expression of which was altered by all toxicants except for PBDE-99; the neurofibromatosis (NF) gene family (Nakamura 1995) which was altered specifically in geldanamycin-treated NCC (NF1 and NF2); and the netrins (Amiel et al. 2008), which were altered in PBDE-99 (NTN4)- and TSA (NTN4 and NTNG)-treated cells.

This analysis on the level of individual genes/PS suggested effects of the toxicants on very different pathways and biological processes (Fig. 4), but such more narrative descriptions are of limited use for toxicological hazard estimates and quantitative approaches. To explore options for quantifications of transcriptome changes, and for comparisons amongst compounds, we employed unbiased approaches to identify disturbed higher-order biological processes.

### Visualization of toxicity patterns based on coordinate regulation of genes involved in joint superordinate *biological processes*

To compare the effects on the transcriptome regulation amongst the different conditions, a multidimensional representation was chosen, as pioneered earlier, e.g., by the ToxPi approach in the ToxCast program (Reif et al. 2013) or by the use of toxicity indices developed on the basis of superordinate biological processes (Waldmann et al. 2014). Such descriptors go beyond the level of individual genes, by quantifying the regulation of entire gene ontologies (Theunissen et al. 2011, 2012; Waldmann et al. 2014) and forming aggregate measures or simplified visualizations.

**Fig. 5:**
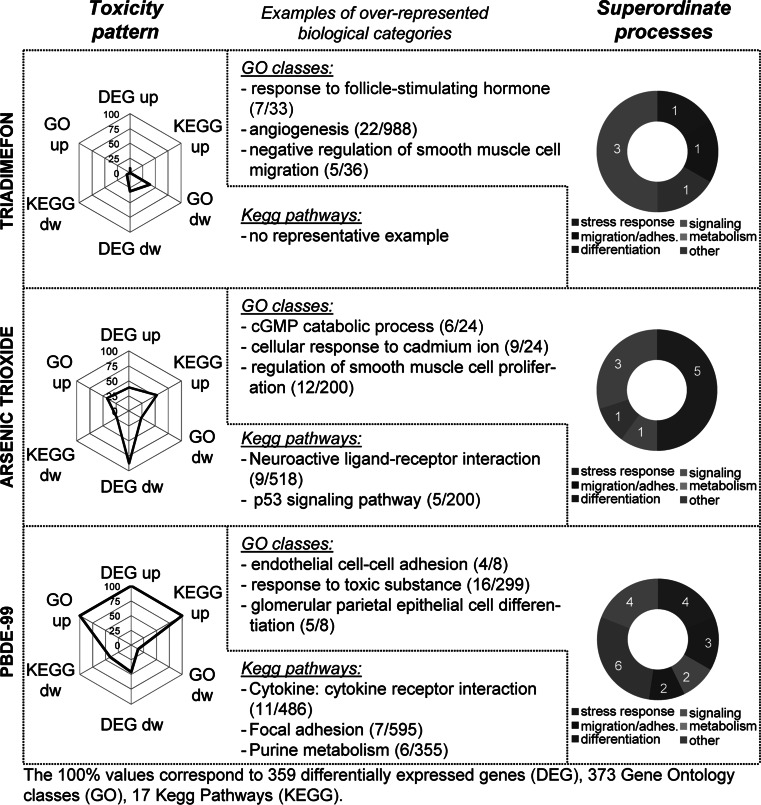
Alteration of superordinate biological processes by environmental pollutants. Graphical display to visualize broad information on biological changes, i.e. the toxicity patterns triggered by the compounds belonging to the class of ‘environmental pollutants’ (triadimefon, arsenic trioxide, PBDE-99): the spider diagrams (*on the left*) indicate normalized numbers of upregulated and downregulated (dw) differentially expressed genes (DEG); of GO terms overrepresented amongst DEG (GO); and of overrepresented KEGG pathways (KEGG). The absolute values used for normalization of each axis are indicated at the *bottom of the figure*. They correspond to the respective highest value for all 6 compounds. The *ring diagrams on the right-hand side* show the relative distribution of 6 superordinate biological processes (stress response, migration/adhesion, metabolism, differentiation, signalling and other) amongst the DEG. The data are based on the counting of non-redundant overrepresented GO terms (detailed table in supplemental material) within each superordinate biological process category (*white numbers*). Identification of overrepresented GO was done on the basis of all DEG (up- and downregulated). In the middle, examples of overrepresented biological categories (KEGG, GO) are shown (with number of regulated and total genes belonging to the specific group) and colour-coded according to the superordinate process

**Fig. 6:**
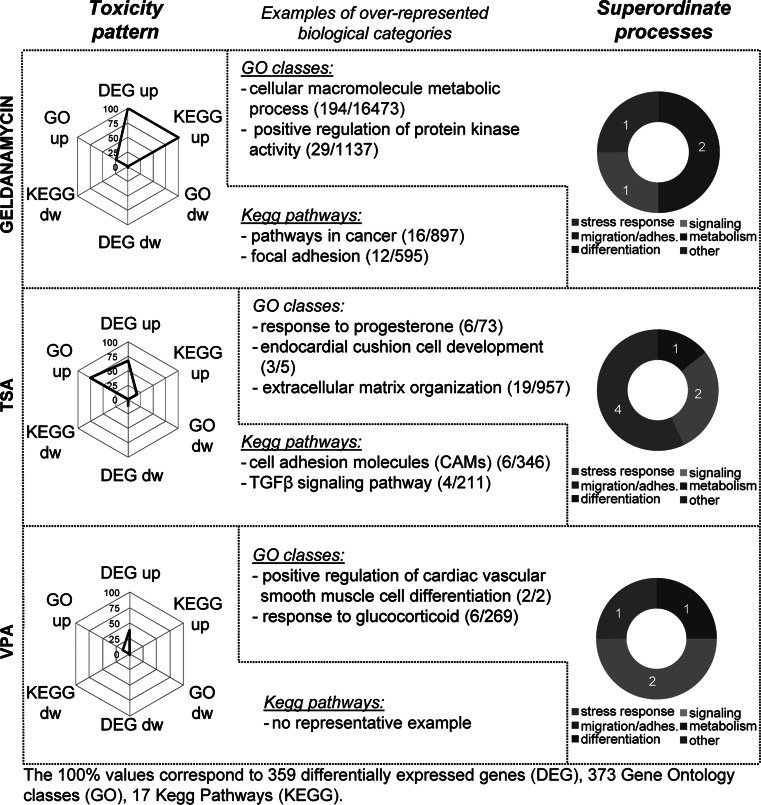
Alteration of superordinate biological processes by drugs. Toxicity patterns triggered by the compounds belonging to the class of ‘drugs’ (geldanamycin, TSA, VPA) are presented as in Fig. 5. The spider diagrams indicate normalized numbers of differentially expressed genes (DEG), GO terms overrepresented amongst DEG (GO) and overrepresented KEGG pathways (KEGG). The absolute values used for normalization of each axis are indicated at the *bottom of the figure*. They correspond to the respective highest value for all 6 compounds. The *ring diagrams* show the relative distribution of 6 superordinate biological processes (stress response, migration/adhesion, metabolism, differentiation, signalling and other) amongst the DEG. In the middle, examples of overrepresented biological categories (KEGG, GO) are shown (with numbers of regulated and total genes belonging to the specific group)

In a first step, the pattern of transcriptome changes was visualized on the basis of six key parameters: the number of up- and downregulated DEG, the number of GO terms enriched amongst upregulated (GO up) and downregulated DEG (GO dw) and the number of KEGG pathways enriched by upregulated (KEGG up) and downregulated DEG (KEGG dw; Figs. 5, 6). To facilitate comparisons, the underlying data were normalized across all compounds to the respective maximum value observed in the whole study. This procedure is similar to the one taken by EPA in their ToxPi approach, and it allows direct comparisons of the patterns observed in the radar plots used here. For instance, it becomes easily evident that TSA was characterized by overrepresented GO terms only amongst its upregulated DEG, while the reverse was observed for triadimefon (only GO terms amongst downregulated DEG). For information beyond an initial overview, all detailed data were compiled in tabular form (Supplementary tables S1-3).

In a second step, the types of biological processes that may be linked to the altered transcriptome of toxicant-treated cells were visualized. For this purpose, all DEG (up and down) were pooled for a given compound. Then, GO term enrichment analysis was performed by a statistical method, the so-called elim algorithm that eliminates ‘redundant’ GO groups, i.e. such GO groups that contain the same genes, but do not provide new information (so-called children GOs or parent GOs of a given term; Alexa et al. 2006). The resultant unique overrepresented GO groups were assigned to five superordinate cell biological processes (Waldmann et al. 2014): stress response, signalling, migration/adhesion, metabolism, differentiation; those that could not be assigned were grouped under ‘other’. The category ‘stress response’ combined all GO terms related to cell death, extracellular stress, inflammation and immunity; the ‘signalling’ category comprised GO terms related to cellular receptors, second messengers, hormones/neurotransmitters and kinase modifications/regulations. The relative contribution of the different superordinate processes to the altered transcriptome response was shown in form of ring diagrams. These allow, for instance, a quick overview that suggests that the response to arsenite (Fig. 5) is dominated by ‘stress responses’, while HDACi responses have a strong ‘signalling’ component (Fig. 6). In case, more detailed information is required, then this can be retrieved from tabular compilations (Supplementary table S4).

Altogether, these overview presentations of transcriptome changes showed at the first glance that the three environmental pollutants had very different effects on neural crest cells, although they all inhibited migration (Fig. 5): triadimefon had a relatively modest influence on the transcriptome, compared to PBDE-99; while the latter one mainly upregulated transcripts, arsenite predominantly downregulated gene activity. However, arsenic trioxide also upregulated some transcripts and these pointed, for instance, to the activation of the p53 pathway, which has been implicated earlier in arsenite toxicity (van Vliet et al. 2007).

The ‘toxicity patterns’ obtained for the drug-like group of test chemicals were characterized by a predominant upregulation of DEG and associated biological processes (Fig. 6). Geldanamycin upregulated genes related to ‘cancer’ KEGG pathways, but also focal adhesion, a process that may be related to the migration-inhibitory activity of this compound in neural crest cells.

The TSA toxicity pattern was mostly characterized by upregulation. Amongst the upregulated DEG, only few KEGG pathways were enrichment (e.g. cell adhesion molecule and TGF-β signalling pathway), but a high overrepresentation of GO terms was found. These included ‘cardiac development’ as well as ‘steroid signalling’, and the latter two were also found for the related compound VPA. In general, VPA triggered a much less pronounced transcriptome response. This is well in line with a relatively specific pharmacological activity of this compound, although it is used at high concentrations in clinical settings, and its mode of action is supposed to affect the chromatin structure of a large number of genes.

### Candidate biomarker identification

**Fig. 7:**
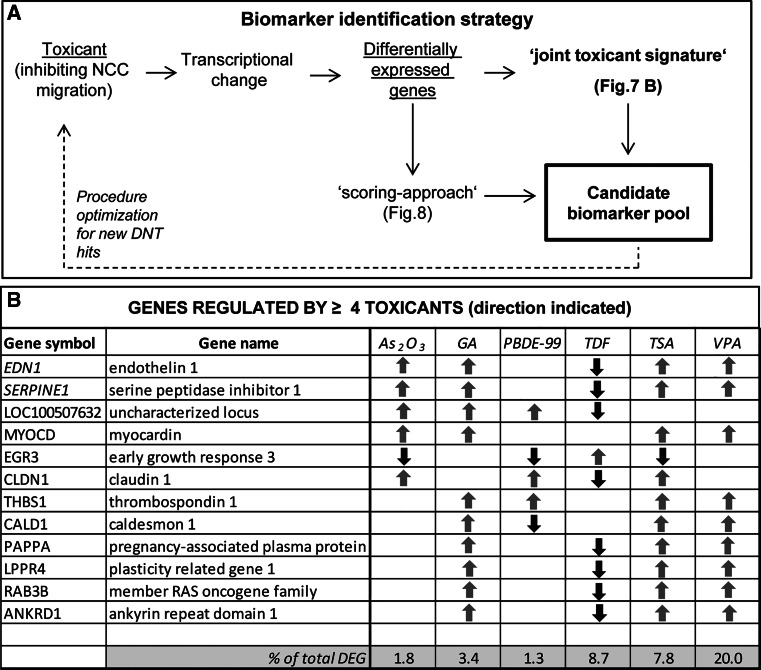
Biomarker identification strategy and joint toxicant signature-derived gene list. **a** Two different approaches were adopted to identify candidate biomarkers in this study: a ‘joint toxicant signature’-approach based on the overlap of DEG amongst the compounds, and a ‘scoring’ approach based on the evaluation and weighting of each gene as described in Fig. 8. **b** The ‘joint toxicant signature’ approach led to the identification of 12 genes which were regulated by ≥4 toxicants; their expression direction is indicated by *red* (downregulation) or *green* (upregulation) *arrows*. At the *bottom*, information is given on how many % of the DEG regulated by each compound are represented in the list of overlapping DEG (colour figure online)

Several hits from the MINC assay had been used here for transcriptome analysis and for a proof-of concept study to blindly predict compounds within a given group. However, for the use of higher numbers of compounds, transcriptome analysis is too expensive and time-consuming. Limitation to particularly informative transcripts, here called ‘candidate biomarkers’ would greatly facilitate alternative approaches by PCR or target-specific next-generation sequencing. Therefore, we used the DEG identified here to define biomarker candidates. We followed two different strategies to select appropriate genes (Fig. 7a).

The first approach was based on the concept that DEG found for several compounds would have a broader applicability (and statistical validity). Therefore, we identified all genes, the expression of which was affected by 4 or more toxicants. We termed this group of 12 genes ‘joint/general toxicant signature’ (Fig. 7b). Two of them (EDN1 and SERPINE1) were shared by 5 compounds, while the other 10 were altered by four toxicants. For the assignment of genes to this group, we did not consider the direction of regulation. For instance, triadimefon downregulated most of the transcripts that were upregulated by the other compounds, and the only of the consensus gene upregulated by triadimefon was downregulated by the other toxicants.

**Fig. 8:**
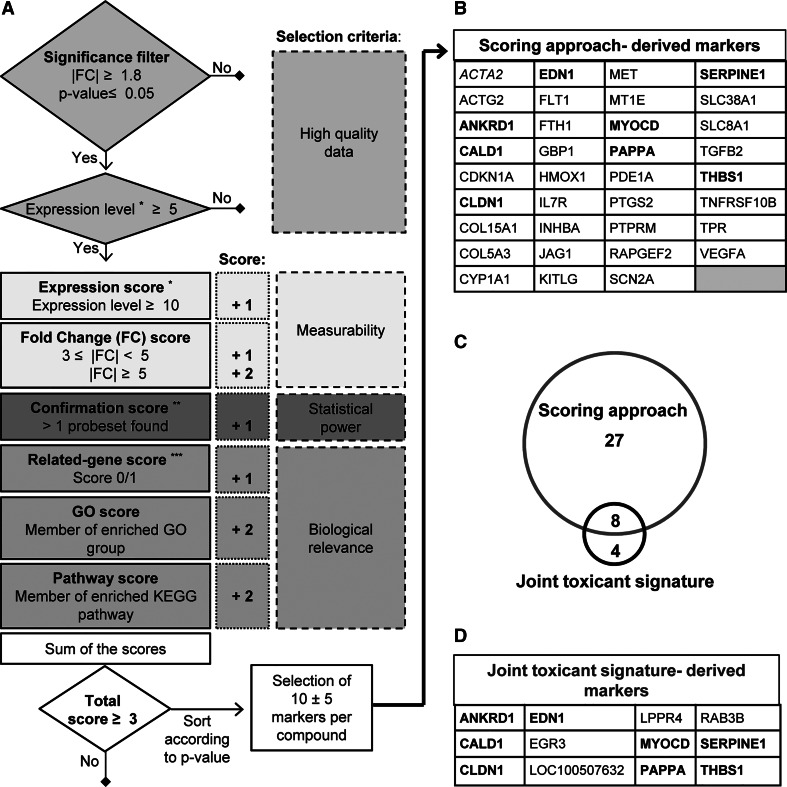
Scoring flow chart and overlap between gene markers identified by the scoring and the joint toxicant signature approaches. **a** The algorithm of the ‘scoring approach’ to identify candidate biomarkers was based on 4 different selection criteria groups: high-quality data, measurability, statistical power and biological relevance (*colour-coded*). Genes were considered for the scoring, when they fulfilled minimum criteria (significance, expression level). Then, scores were given for each gene according to criteria as indicated. Genes with a score ≥3 were shortlisted and used for further selection of a final list of candidates with particularly low *p* values and balanced across the six toxicants. **b** This approach led to the selection of 35 candidate biomarkers. Those amongst them also found by the ‘joint toxicant signature’ approach (Fig. 7) are marked in *pink*. **c** The overlap of different types of candidate biomarkers is shown in a Venn diagram, **d** and, for clarity reasons, the ‘joint toxicant’ markers are explicitly displayed, with the overlapping ones also marked in *pink*. *The expression level is the absolute fluorescence value of each probe set (after RMA normalization) which ranged on a scale from 3 to 15. **The ‘confirmation score’ indicated that >1 PS was regulated (*in the same direction*) for a given gene. ***The ‘related-gene score’ was applied when, for a given gene, additional related genes were found in the DEG list (belonging to same family or sharing same receptor) (colour figure online)

Our second approach to identify a pool of candidate biomarkers followed a ‘scoring approach’ (Fig. 7a). We considered each gene that was regulated by one of the six toxicants and then assigned it a certain importance score according to a filtering and ranking algorithm (Fig. 8a). Initially, DEG had to fulfil three minimum requirements to be considered for scoring: a |FC| ≥ 1.8, a *p* value ≤0.05 (corrected for false discovery rate) and a control expression level clearly above the microarray noise level, i.e. a fluorescence value of at least 5 after RMA normalization (on a log2 scale ranging from 3 to 15).

Genes were then evaluated based on three further selection criteria (measurability, statistical power and biological relevance) and scored accordingly. For instance, a scoring point was assigned to a given gene, when additional related genes (e.g. belonging to same family or sharing same receptor) were found in the DEG list. We felt that such genes would have a higher value as biomarkers, as they reflect a regulation mechanism (coordinated regulation of functionally linked genes) going on in treated cells. For the same reason, additional scores were given to genes which were members of enriched GO classes and KEGG pathways.

**Fig. 9:**
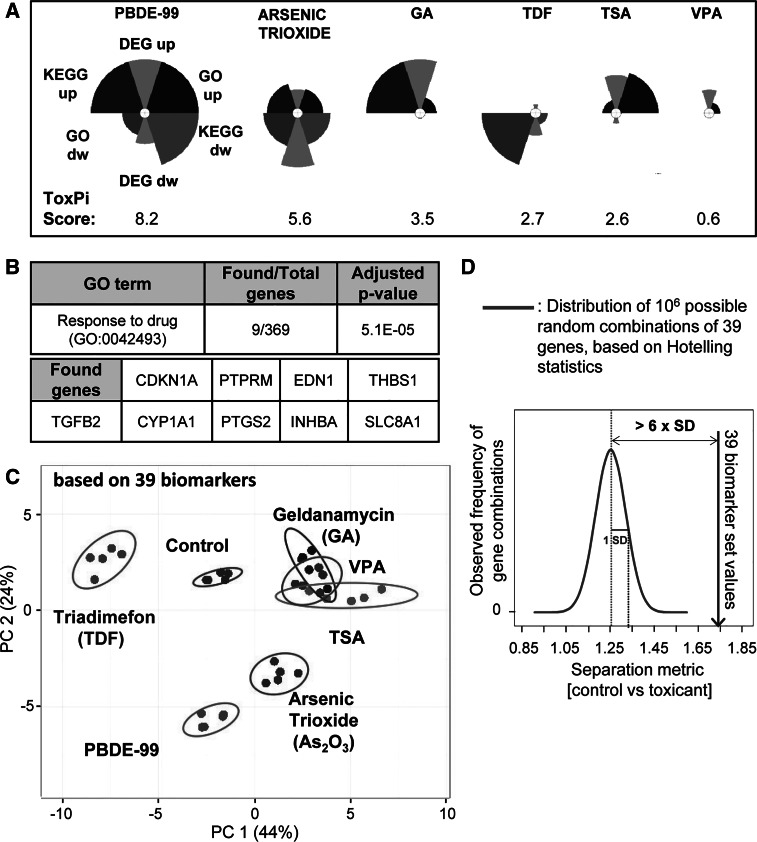
Summary of observations and biomarker set characteristics. **a** The ToxPi diagrams were built using the ToxPi GUI (Toxicological Priority Index graphical user interface) software tool developed in the ToxCast project (Reif et al. 2013). The numbers of up- and downregulated DEG, enriched GO classes and KEGG pathways were normalized (range from 0 to 1) to the respective highest values (359 DEG up, 313 DEG dw, 373 GO up, 146 GO dw, 17 KEGG up and 7 KEGG dw) for each category, across all the compounds. Each slice of the diagram contains several types of information: the distance from the centre, proportional to the normalized value of the composing that slice; the width (in radians) indicates the relative weight of that slice in the overall ToxPi calculation. In our analysis, double weight was given to the KEGG and GO slices (80 % of the total), while DEG slices contributed with 20 % of the weight. The calculated ToxPi score is indicated for each compound (under each diagram) as parameter for potential developmental toxicity. **b** GO enrichment analysis of 39 biomarker candidates was performed using the GO elim algorithm. The most significant GO class is listed together with the genes, which contributed to its enrichment. **c** A 2D-PCA plot was constructed for the six toxicants, based on the expression data for the 39 candidate biomarkers. The values for each gene were calculated as the median value amongst the probe sets specific for that particular gene. The analysis was performed using the freely available Web tool ClustVis (http://biit.cs.ut.ee/clustvis). The ellipses show the 95 % CI. **d** From the pool of differentially expressed genes in NCC treated with toxicants, 39 samples (= genes) were drawn at random 1 million times. The separation strength (extent of separation of controls from the group of toxicants of these samples) was calculated (*x* axis; parameter ranges from 0 to 2.45), and the distribution was plotted. For comparison, the separation strength of the here selected biomarker set is indicated

Finally, the genes with a score ≥3 were shortlisted, and this pool of markers was saved for potential later use (Supplementary table S5). For extraction of a reasonably small number of candidate biomarkers from this list, 8–12 genes per compound were selected manually (non-mathematical approach) in a way to (1) ensure a reasonable balanced across the six toxicants, (2) give preference to genes with high scores and (3) favour low *p* values. This resulted in a final group of 35 candidate biomarkers (Fig. 8b). Notably, eight genes of this group overlapped with the biomarkers that were selected based on their role as ‘joint toxicant signature’ (Fig. 8c, d). Thus, this final part of the study yielded altogether a pool of 39 candidate biomarkers that are of interest for further evaluation in a larger toxicant screen to predict neural crest functional toxicity or to allow grouping of toxicants according to shared mechanisms/biomarker signatures (Fig. S2). A statistical evaluation of this biomarker set indicated that it showed differences between the toxicants, was clearly related to the cell biology of drug response and performed much better than randomly selected sets of markers (Fig. 9).

## Discussion

In the present study, we analysed transcriptome changes triggered by six hits of the ESNATS test battery. The compounds examined here had been identified as potential developmental toxicants because of their ability to inhibit NCC migration at non-cytotoxic concentration and the working procedure of the ESNATS test battery (Zimmer et al. 2014; Dreser et al. 2015) required further characterization, including transcriptome mapping. The transcriptome data obtained here allowed the separation of the toxicants on a principal component map. Moreover, they allowed the assignment of unknown samples to the known set of compounds, based on a support vector machine classification algorithm. Finally, the types of biological changes, indicated by the altered transcriptomes, were visualized in diagrams focusing on altered superordinate biological processes.

In this study, three questions were asked to explore the usefulness of transcriptome changes for a toxicant test battery. The first question addresses a key issue of toxicogenomics in general: can transcriptome profiles be used to identify hazardous compounds? Previous studies have shown that this is a highly demanding challenge and that a lot need to be learned on better design and evaluation of transcriptomics studies for such purposes (Thomas et al. 2013; Grinberg et al. 2014; Bourdon-Lacombe et al. 2015; El-Hachem et al. 2015). Thus, this large issue needs to be approached in smaller steps. A main issue that became evident in our study is the large heterogeneity in transcriptome responses triggered by compounds that all affect the same functional endpoint (NC migration). One reason may be that migration of cells is such a complex endpoint that compounds with very diverse modes of action can affect it; i.e. very different molecular initiating events will affect the same adverse outcome. This precludes any simple type of analysis, such as the identification of a joint gene derangement pattern across all compounds. However, on the positive side, toxicants known to share a known mode of action, such as VPA and TSA, also showed a similar transcriptome response. This implies that it may be possible to use transcriptome responses for toxicological grouping of compounds; i.e. that an unknown compound may be assigned to a group of already known toxicants based on shared transcriptome profiles. This would be an expansion of the read-across approach, away from structure-based algorithms to the incorporation of variable biological information (Low et al. 2013; Patlewicz et al. 2014; Bal-Price et al. 2015; Berggren et al. 2015). The basis for this was explored here in a small pilot study to see whether an unknown chemical could be assigned within a small, but diverse group of toxicants to its most related compound. The blind assignment of six compounds to the six known compounds worked surprisingly well, given the fact that only three microarrays per compound were used to build the classifier.

Given the situation that in vivo testing for developmental toxicity, and especially developmental neurotoxicity has serious issues concerning species extrapolation and sensitivity (van Thriel et al. 2012; Smirnova et al. 2014), there is a large need to consider new approaches for risk assessment or at least filtering of relevant compounds for further testing. One such approach is the consideration of key biological processes, such as neural crest migration (Bal-Price et al. 2012; Kadereit et al. 2012) that can be tested in appropriate in vitro systems. Compounds that affect such key biological processes could be further investigated for transcriptome changes in the respective system, and this information may then be used to better define the mode of action, but also to read across to other compounds with known in vivo toxicities. Similar approaches have been tried with promising results across largely different systems, not only based on human cells, but, e.g., also using model organisms such as zebra fish (Hermsen et al. 2013). A more radical future way would be to compare toxicants based on their transcriptome profiles in well-characterized test systems rather than on their effects in animals. One condition for this is a high level of test system characterization and quality control (Leist et al. 2010, 2012b; Crofton et al. 2011; Coecke et al. 2005). Beyond this, a large knowledge base needs to be collected on whether toxicogenomics signatures really can predict toxicity in a given test system. Until now, only few functional developmental toxicity assays have been evaluated in this direction, and it is unclear whether, e.g., neurite growth or neurite degeneration assays (Volbracht et al. 1999; Stiegler et al. 2011; Krug et al. 2014) fulfil such conditions. Also for general neurodegeneration assays, there is still little information, as transcriptome information needs to be obtained during a phase prior to cell death. Moreover, if generated from complex models, such as cocultures (Alepee et al. 2014; Efremova et al. 2015), it needs to be distinguished from the generalized inflammatory response (Falsig et al. 2004; Falsig et al. 2006). If such conditions are fulfilled, as recent studies have shown in the case of damage triggered by MPP+ in human neurons, then toxicogenomics information can yield surprising new information and mechanistic insight (Krug et al. 2014) and a related in vivo study also revealed hitherto unsuspected genetic regulations (Maertens et al. 2015; Rahnenfuhrer and Leist 2015).

A second question addressed in this study is how primary transcriptome information; i.e. long lists of differentially expressed genes can be reduced to a format that is easier to handle and that can be used for toxicological purposes. Classical toxicology has worked well with semiquantitative information that is judged for its significance by experts and that requires careful consideration of many modulatory and circumstantial factors. For instance, staining of histological slides may indicate cellular changes characterized by eosinophilia, hypertrophy and lipid droplet accumulation, and experts have to decide on the type and level of hazard this indicates or whether this is rather an insignificant or adaptive change. A similar system of several dozen to hundreds of categories is required for toxicogenomics, while dealing with 25,000 individual genes will not be possible. One composite measure is the number of DEG. It appears evident that the information content of such an endpoint is relatively low, although there is a high likelihood that compounds that do not deregulate any gene are relatively harmless, and chemicals that deregulate very large numbers of genes may be problematic. More information may be obtained from the examination of biologically linked gene networks, i.e. genes belonging to one GO group of KEGG pathway. An increasing number of overrepresented GO terms/KEGG pathways (or other biological motives) amongst the DEG would indicate a specific regulation of genes belonging to a certain cell function as opposed to random gene regulations. A summary of the changes across all study compounds can be obtained from such measures very quickly, e.g., in the form of ToxPi diagrams (Reif et al. 2013), or the associated ToxPi score (Fig. 9a).

Even more information may be gained by looking exactly into which pathways (KEGG) are regulated or which specific groups the regulated genes belong to. However, a compromise has to be found between the detail of information and the simplicity of an initial toxicological statement. The chosen solution was to show only numbers of superordinate biological processes together with few examples and a rough classification. In addition to this coarse-grained initial information layer, supplementary information can then answer details, where required.

Even for such standard approaches, some decisions have to be taken. They include the statistical criteria used to define the DEG, but also involve issues such as the separate or combined treatment of up- and downregulated genes. We decided here to identify overrepresented GO and KEGG separately in the two groups of genes. With this procedure we followed an established routine that was chosen to facilitate comparisons amongst different conditions, such as different concentrations of one compound, or one concentration of different compounds, or different exposure times of one compound (Krug et al. 2013b; Balmer et al. 2014; Waldmann et al. 2014; Rempel et al. 2015). When defining intersection between conditions, we felt that it is important to consider the direction of regulation of a gene and also to mine the genes accordingly for overrepresented biological themes. A different situation is encountered, when no such comparisons are intended and when different directions of regulation, e.g., within a GO group make biological sense (e.g. ‘positive regulation of apoptosis’ involves upregulation of apoptosis inducers (BCL-2, caspases) and downregulation of inhibitors (BAX, IAPs, HSP70; Latta et al. 2000; Gerhardt et al. 2001; Hansson et al. 2003). For this reason, GO analysis to identify superordinate biological processes was performed differently than for the general data exploration.

The third major question was directed to the identification of biomarker candidates that would allow a simplified approach, compared to whole-genome transcript profiling. The term ‘biomarker’ has a wide range of implications and uses and therefore requires some definition in the context of our study. Very strict definitions are found in the field of predictive medicine, or in toxicology in the form of a biomarker of toxicity (BoT) that is required to have a high predictive value and to show some causal relationship with the adverse outcome (Blaauboer et al. 2012) or a toxicity pathway (Leist et al. 2008). At the other end of the spectrum, biomarkers are simply seen as any endpoint that changes in a test system upon exposure to a test compound. An approach somewhere in between these extremes is to define biomarkers as preselected endpoints with a certain information value concerning the study purpose (e.g. biomarker of exposure or biomarker as part of a predictive gene signature), but not necessarily linked to the mechanism of action of a compound. A good example for this type of approach is the GARD assay for skin sensitization (Johansson et al. 2013, 2014), in which first whole-transcriptome data were obtained on skin sensitizers and negative controls, and then a statistics-based algorithm was applied to select the set of genes (biomarkers) that was most useful as classifier. Similar approaches have been taken to add biological information for read-across, for instance, in the SEURAT-1 research project on prediction of cosmetics toxicity (Gocht et al. 2015) or based on the TG-GATES transcriptome data (Low et al. 2013). We provided here a basis for such a latter approach by selecting a small number of genes from all the DEG of the study.

These were termed here ‘candidate biomarkers’ as more work is required to qualify them as ‘real’ biomarkers at a confidence level of, e.g., the GARD assay. An immediate usefulness is suggested by analysis of overrepresented GO terms amongst the 39 selected genes: the most significant enriched GO class was ‘response to drug’ (Fig. 9b). Moreover, the chosen set of genes provided a good basis for separation of the study compounds from control cells (Fig. 9c, Fig.S3, Supplementary table S6). To obtain an idea on the performance of the selected biomarkers, relative to random sets of biomarkers, we use here a relatively simple and transparent approach to define ‘separation strength’, and we explored this separation strength of our biomarker set, when compared to one million sets, randomly chosen from the pool of all regulated probe sets of this study. The 39 biomarker sets were by far superior to random sampling (99–100th percentile of the distribution, with 6 standard deviations distance to the means; *p* < 10^−6^; Fig. 9d), which confirms the overall usefulness of our selection approach. We are aware of the relatively extreme assumptions we had to make (e.g. statistical independence of the endpoints), and that separation strength may be defined in many other ways. However, we hope that this initial attempt will trigger more work in this area, allowing establishing criteria for separation strength of a set of biomarkers (as opposed to a classifier formula). In the future, a consensus will then need to be reached on statistical approaches to judge the quality of a given biomarker set relative to randomly selected sets.

At present, the explorative approaches discussed here qualify our small set of candidate biomarkers for further exploration and possible substitution of the full microarray approach by a cheaper and faster technology. Three major steps will have to be taken towards this objective in the future: (1) the marker gens would require confirmation by the alternative analytical technology chosen (e.g. PCR) on the set of study compounds used here; (2) then, they would need to be tested for their usefulness on another set of compounds (Leist et al. 2010; Crofton et al. 2011); (3) and finally, the time and concentration relationship of marker changes would need to be correlated with the functional toxicity (inhibited migration) in the MINC assay.

In summary, this study added information on ESNATS test battery hits and provided a case study on the predictive value of toxicogenomics by showing that chemicals can be predicted, based on their transcriptome changes, at least within a smaller group. Extension to more compounds will be necessary. Moreover, tools were developed to visualize transcriptome changes and to provide at least semiquantitative data on the extent and type of transcriptome derangement. Finally, two different approaches were combined to preselect biomarkers that are still able to separate the compounds and that will require further evaluation for their application in predictive toxicology.

## Electronic supplementary material

Supplementary material 1 (XLSX 3651 kb)

Supplementary material 2 (XLSX 6239 kb)

Supplementary material 3 (XLSX 4306 kb)

Supplementary material 4 (XLSX 4476 kb)

Supplementary material 5 (XLSX 955 kb)

Supplementary material 6 (XLSX 969 kb)

Supplementary material 7 (PDF 880 kb)

## Abbreviations

BH: Benjamini–Hochberg method for *p* value adjustment for multiple comparisons
BOT: Biomarker of toxicity
DEG: Differentially expressed genes
ESNATS: Embryonic stem cell-based novel alternative tests
FC: Fold change
GA: Geldanamycin
GO: Gene ontology
HDACi: Histone deacetylase inhibitor
hESC: Human embryonic stem cell
HSP: Heat-shock protein
KEGG: Kyoto encyclopaedia of genes and genomes
LUHMES: Lund human mesencephalic
MINC: Migration of neural crest cell
NC: Neural crest
NSPC: Neural stem and progenitor cells
PBDE: Polybrominated diphenyl ether
PCA: Principal component analysis
PCB: Polychlorinated biphenyl
PS: Probe set
SVM: Support vector machine
TDF: Triadimefon
TSA: Trichostatin A
VPA: Valproic acid
