# Supplementary material for: Identification of transcriptome signatures and biomarkers specific for potential developmental toxicants inhibiting human neural crest cell migration
Source: Arch Toxicol. 2015 Dec 26;90:159–80. doi: 10.1007/s00204-015-1658-7 (PMC4710658; doi:10.1007/s00204-015-1658-7)
Supplement: Supplementary file 7 — Supplementary material 7 (PDF 880 kb) [file 204_2015_1658_MOESM7_ESM.pdf]

Supplemental Material

Identification of transcriptome signatures and biomarkers specific for potential developmental toxicants inhibiting human neural crest cell migration

Giorgia Pallocca, Marianna Grinberg, Margit Henry, Tancred Frickey, Jan G. Hengstler, Tanja Waldmann, Agapios Sachinidis, Jörg Rahnenführer, Marcel Leist

Table of contents

- **Overview on content of supplemental-tables folder** **page 2**

- **Supplemental Material, Figure S1** **page 3**
  Evaluation of theSVM-based classifier.

- **Supplemental Material, Figure S2** **page 4**
  "Scoring approach" applied to the candidate biomarker genes.

- **Supplemental Material, Figure S3** **page 5**
  Expression pattern of the candidate biomarker genes among the different exposure conditions.

Overview on content of supplemental-tables folder

Suppl. Table S1 - *Differentially regulated probesets in neural crest cells (UNK2 system) after exposure to six different conditions*. Data of 5 independent experiments on Affymetrix microarrays are displayed including gene names, fold of change and adjusted p-value, per each exposure scenario.

Suppl. Table S2 - *Overrepresented GO classes in the six test conditions.* The table shows the list of the gene onthology classes (GO) that were enriched in sets of differentially expressed genes (identified by microarray analysis). Data are displayed including the name and the ID of the GO class, the corresponding number of total genes belonging to the GO class, the number of found DEG belonging to the GO class, and the adjusted p-values, per each exposure scenario.

Suppl. Table S3 - *Overrepresented KEGG pathways in the six test conditions.* KEGG pathways (KEGG) enriched in populations of differentially expressed genes (identified by microarray analysis) were determined. Data are displayed including the name and the ID of the KEGG pathway, the corresponding number of total genes belonging to the KEGG pathway, the number of found DEG belonging to the KEGG pathway, and the adjusted p-values, per each exposure scenario.

Suppl. Table S4 - *Overrepresented GO classes using up- and down-regulated probesets and respective classification in superordinate processes.* The table shows the gene onthology classes (GO) that were enriched in populations of differentially expressed genes (using as input up- and down-regulated probesets) and their distribution in superordinate processes. Data are displayed including the name and the ID of the GO class, the corresponding number of total genes belonging to the GO class, the number of found DEG belonging to the GO class, the adjusted p-values and the superordinate process which they belong to.

Suppl. Table S5 - *Individual scores for candidate biomarker- gene list.* The table shows the singular scores assigned to each gene selected by the flow-chart in Fig.8. Data are displayed including compound and gene name, and the values of expression-, fold change (FC)-, confirmation-, related-gene-, GO and pathway- scores.

Suppl. Table S6 - *Median of the absolute expression among the probesets of the selected list of candidate biomarker genes.* The table shows the median of the absolute expression among the probesets of each gene selected by the flow-chart in Fig.8. Data are displayed including the biomarker gene list and the values of absolute espression expressed as median among the probeset.

Supplemental material, Fig.S1:
Evaluation of the SVM-based classifier

A

Truth \ Predicted as	Prediction frequency [%] As_2O_3	GA	PBDE-99	TDF	TSA	VPA
As_2O_3	100.00	0.00	0.00	0.00	0.00	0.00
GA	0.00	99.95	0.00	0.00	0.05	0.00
PBDE-99	0.00	0.00	100.00	0.00	0.00	0.00
TDF	0.00	0.00	0.00	100.00	0.00	0.00
TSA	0.00	0.00	0.00	0.00	25.60	74.40
VPA	0.00	0.00	0.00	0.00	29.95	70.05

B

Blind Repl.	Probabilities: Best prediction	2nd best prediction	Truth
b1	PBDE-99 (0.50)	VPA (0.18)	*PBDE-99*
b2	PBDE-99 (0.54)	As_2O_3 (0.13)	*PBDE-99*
c1	*TDF* (0.49)	VPA (0.18)	*TDF*
c2	*TDF* (0.45)	VPA (0.25)	*TDF*
d1	GA (0.67)	As_2O_3 (0.10)	*GA*
d2	GA (0.70)	As_2O_3 (0.10)	*GA*
e1	VPA (0.56)	GA (0.13)	*VPA*
e2	VPA (0.42)	GA (0.31)	*VPA*
f1	As_2O_3 (0.40)	VPA (0.18)	As_2O_3
f2	As_2O_3 (0.50)	VPA (0.16)	As_2O_3

Supplemental material, Fig.S1:
The support vector machine (SVM)-based classifier as described in Fig.2 was used to predict different scenarios. A A simulation study of the SVM-based classifier (Fig.2) was performed: three replicates (of 5) per compound were randomly chosen to form the training set and to build the classifier. The identity of the remaining 2 replicates (testing set) was then predicted. The procedure was reiterated for 1000 times. Finally, the best predictions were summed (considering together the replicates of the same compound) and normalized (n° prediction/ 2000 * 100). B The compound TSA was excluded from the training and testing set. The 100 probe sets with highest variance (“100 PS”) within the training set were newly identified, and used to build a new classifier. The best and second best predictions, based on a support vector machine approach (indicated as relative probability in the brackets), are listed for each blind replicate (first column). The real identity of the samples (truth) is indicated in the last column.

Supplemental material, Fig.S2:
"Scoring approach" applied to the candidate biomarker genes.

	Score						
	TDF	PBDE-99	TSA	VPA	As_2O_3	GA	TOT
SERPINE1	5	nd	3.5	2	5	6	21.5
CLDN1	2	6	5	nd	3	nd	16
THBS1	nd	5	5	2	nd	4	16
EDN1	3.5	nd	3	2	3	3	14.5
PTGS2	nd	7	nd	nd	7	nd	14
TGFB2	5	nd	5	nd	nd	4	14
TPR	nd	7	nd	nd	nd	7	14
ACTA2	5	5	nd	3	nd	nd	13
MT1E	nd	3	0	3	6	nd	12
MYOCD	nd	nd	2	3	2	5	12
PDE1A	nd	6	nd	nd	6	nd	12
ANKRD1	4	nd	3	2	nd	2	11
PAPPA	3	2	4	nd	nd	2	11
RAB3B	3	nd	3	2	nd	3	11
SLC8A1	nd	2	nd	nd	nd	8	10
CALD1	nd	5	0	3	nd	1	9
FLT1	nd	nd	3	nd	nd	6	9
INHBA	nd	nd	3.5	2	nd	3	8.5
COL5A3	nd	8	nd	nd	nd	nd	8
IL7R	3	nd	5	nd	nd	nd	8
LPPR4	3	nd	2	2	nd	1	8
COL15A1	nd	7	nd	nd	nd	nd	7
CYP1A1	nd	7	nd	nd	nd	nd	7
GBP1	4	3	nd	nd	nd	nd	7
HMOX1	nd	nd	nd	nd	7	nd	7
PTPRM	nd	7	nd	nd	nd	nd	7
SCN2A	nd	nd	nd	3	4	nd	7
VEGFA	nd	7	nd	nd	nd	nd	7
CDKN1A	nd	nd	nd	nd	6	nd	6
FTH1	nd	nd	nd	nd	6	nd	6
MET	nd	nd	nd	nd	nd	6	6
RAPGEF2	nd	nd	0	nd	nd	6	6
SLC38A1	nd	nd	nd	nd	nd	6	6
JAG1	nd	2	3.5	nd	nd	nd	5.5
KITLG	nd	nd	nd	nd	5.5	nd	5.5
TNFRSF10B	nd	nd	nd	nd	5.5	nd	5.5
EGR3	1	2	0	nd	2	nd	5
ACTG2	4	nd	nd	nd	nd	nd	4
LOC100507	nd	3	nd	nd	0	1	4

Supplemental material, Fig.S2:
List of the candidate biomarker genes and their respective scores calculated per each compound using the "scoring"- algorithm showed in Fig.8A.

Supplemental material, Fig. S3:
Expression pattern of the candidate biomarker genes among the different exposure conditions

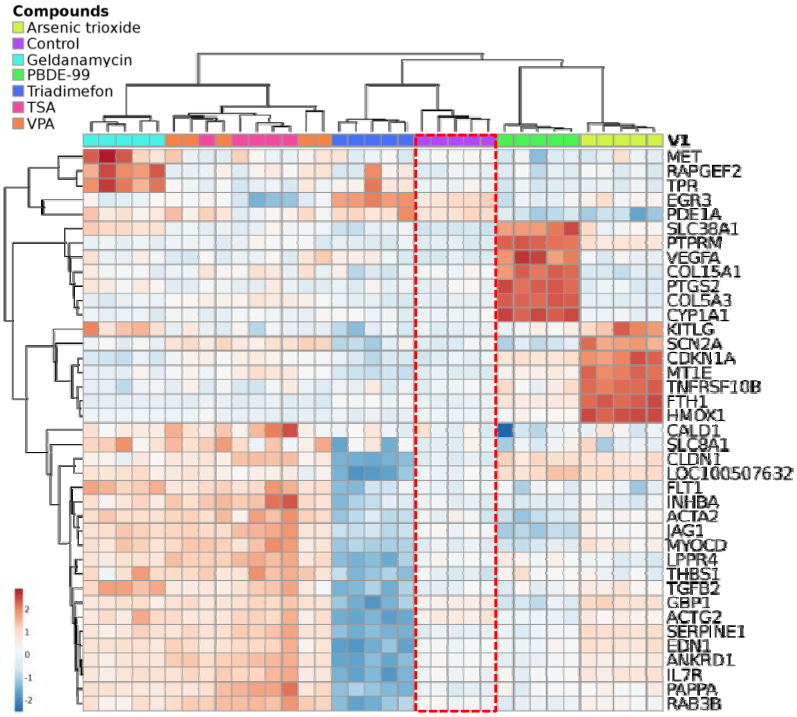

Supplemental material, Fig.S3:
Heat map showing the expression values (expressed as median among the probesets of an individual gene) of each candidate biomarker gene in each exposure condition. Control group indicated by red line.
